# Genetic diversity of two color morphs of Northern snakehead (*Channa argus*) unveiled by the mitochondrial DNA D-loop region

**DOI:** 10.1080/23802359.2022.2029601

**Published:** 2022-04-01

**Authors:** Wei Fan, Lu Zhang, Jian Su, Yu Luo, Xiao-Lei Jiao, Zhi-Peng Huang, Han Zhao, Zhong-Meng Zhao, Yuan-Liang Duan, Qiang Li, Jun Du, Ting Zhuo, Quan-Sen Su, Jun Wu, Jian Zhou

**Affiliations:** a Neijiang Academy of Agricultural Sciences, Neijiang, China; b Fishery Institute of the Sichuan Academy of Agricultural Sciences, Chengdu, China

**Keywords:** *Channa argus*, D-loop region, Genetic diversity

## Abstract

To analyze the genetic background of ‘white’ type Northern snakehead (*Channa argus*), and provide atheoretical basis for breeding of *C. argus*, the investigation of genetic diversity and population structure were investigated based on the complete sequences of mitochondrial DNA D-loop region for three cultured ‘white’ type *C. argus* populations, and four ‘bicolor’ type *C. argus* populations were used to compare with them; 28 mutation loci and 30 haplotypes were found in the D-loop sequence of all individuals with a total length of 907 bp. The highest haplotype diversity (*H_d_*) and nucleotide diversity (*P_i_*) in the ‘white’ type *C. argus* populations were 0.505 and 0.00057, respectively, which lower than those in the ‘bicolor’ type *C. argus* populations (*H_d_* = 0.911, *P_i_* = 0.00326). Population differentiation values (*F*_ST_) show that the four ‘bicolor’ type *C. argus* populations had obvious genetic differentiation (*Fst*: 0.21902–0.49428. *p* < 0.01), but not in the three ‘white’ type *C. argus* populations (*Fst*: −0.00571 to 0.07261. *p* > 0.05). The phylogenetic tree and Median Joining (MJ) network showed that the genetic distance among ‘white’ type *C. argus* populations is very close. Therefore, much attention should be paid to protecting population genetic diversity and avoiding inbreeding in the breeding of ‘white’ type *C. argus*.

## Introduction

The Northern snakehead (*Channa argus*) is the most widely distributed and most prolific species in the Channidae (Wang et al. 2020). There are two distinct color morphs of *C. argus*; The ‘bicolor’ type *C. argus* exhibits a decorative pattern of alternate black and white color, and it is widely distributed in China. The ‘white’ type *C. argus* is white without any blotches, and it is only found in Jialing river (Su et al. 2018; Zhou et al. 2021). The two color morphs of *C. argus* has been classified as two distinct species (Kimura 1934), and the ‘white’ type was written as ‘*Opniocepnalus Argus var*’ in some studies (Li et al. 2016; Zhou et al. 2018). However, some studies compared the morphological characteristics, lactate dehydrogenase, esterase isozyme, chromosome type, and mitochondria of the two color morphs of *C. argus*, and they showed that the ‘white’ type *C. argus* could not be divided into a subspecies and should be regarded as an albino variant of the ‘bicolor’ type *C. argus* (Wang et al. 1992; Zhou et al. 2021). However, the contents of nutrients such as crude protein and polyunsaturated fatty acid in the muscle of ‘white’ type *C. argus* were higher than ‘bicolor’ type *C. argus* (Zhou et al. 2018), and the ‘white’ type *C. argus* is very popular among consumers because of its good taste, medicinal, and ornamental values (Su et al. 2018).

Since the 1990s, artificial breeding of wild ‘white’ type *C. argus* populations has been conducted by harvesting from the Jialing River (Liu 1997). The studies on ‘white’ type *C. argus* were mainly focused on breeding technology (Su and Xiong 2001), culture model (Su et al. 2019), chromosome karyotype (Li et al. 2016), disease (Mou et al. 2014; Wu et al. 2018), and nutritional composition (Deng et al. 2019), but there are few studies on the genetic diversity of ‘white’ type *C. argus*. In recent years, the scale of ‘white’ type *C. argus* cultivation has increased (Su et al. 2018). To protect and utilize the resources of ‘white’ type *C. argus*, it is necessary to investigate the genetic diversity and population structure of ‘white’ type *C. argus*.

The mtDNA D-loop region contains conserved fragments, and it is also the region with the largest variation in sequence and length (Chen et al. 2011). The mtDNA D-loop sequences have been widely used in the study of genetic differentiation, genetic structure, and variation in fish, such as the genetic structure and variation in the wild and breeding populations of Chinese carp (Liu et al. 2017), genetic diversity and population structure of endangered *Clarias magur* (Das 2020), and the genetic variation of *Ptychidio jordani* (Peng et al. 2020). The mtDNA D-loop region has been used to analyze the genetic diversity and population structure of ‘bicolor’ type *C. argus* populations in different regions, such as the Huaihe River (Xiao et al. 2013), Baiyangdian and Dongting lake (Dong et al. 2014), the rivers in Shangxi, Henan, Luoyang, and Jiangsu province (Zhou et al. 2017). Many studies have been reported on the genetic diversity and population structure of ‘bicolor’ type *C. argus*, but few studies have been reported on ‘white’ type *C. argus* (Zhou et al. 2017, 2021). Thus far, little is known about the genetic background of ‘white’ type *C. argus*; therefore, it is essential to study the mtDNA D-loop region to understand the genetic diversity and population structure of ‘white’ type *C. argus* populations.

In this study, the genetic diversity and population structure were evaluated based on the complete sequences of mitochondrial DNA D-loop region for three cultured ‘white’ type *C. argus* populations. Meanwhile four ‘bicolor’ type *C. argus* populations were used for genetic comparison with the ‘white’ type *C. argus*, with the aim of provide a theoretical basis for breeding, protection of fishery resources, and sustainable development.

## Materials and methods

### Study area

**Figure 1.**
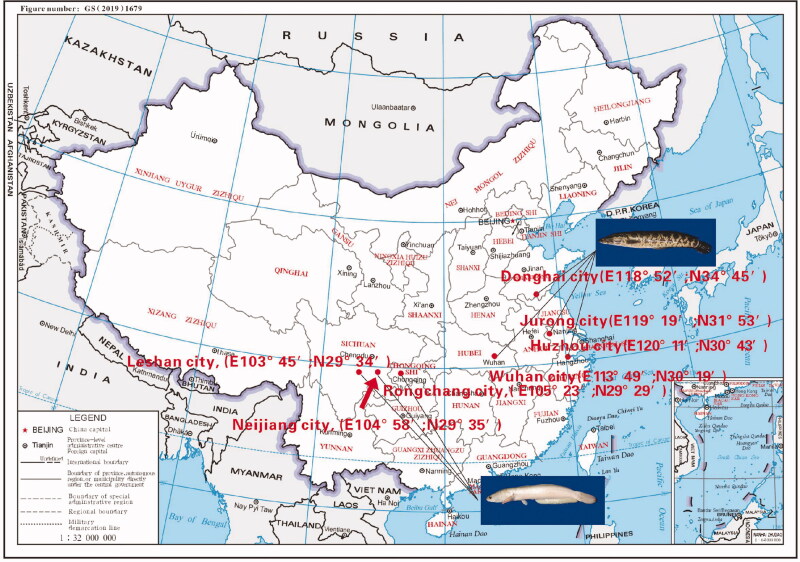
Schematic diagram of the study area.

A total of 350 individuals of two color morphs *C. argus* were collected from different geographical locations (Figure 1); 150 individuals of ‘white’ type *C. argus* were collected, among which 50 individuals each were obtained from Rongchang city (RC), Neijiang city (NJ), and Leshan city (LS). Besides, 200 individuals of ‘bicolor’ type *C. argus* were collected, among which 50 individuals each were obtained from Donghai city (DH), Wuhan city (WH), Jurong city (JR), and Huzhou city (HZ).

### Sample collection

A part of caudal fin was collected from each individual, preserved in 95% alcohol and stored at −20 °C in the herbarium of Fishery Institute of the Sichuan Academy of Agricultural Sciences. The genomic DNA was extracted using an Ezup Column Animal Genomic DNA Purification Kit (Sangon Biotech Co., Ltd., Shanghai, China) and stored at −20 °C.

### PCR amplification and sequencing

The primers were designed using Primer 5.0 (Zhai et al. 2008). According to the full-length mitochondrial sequence of *C. argus* (GenBank NC015191.1), the upstream primers and downstream primers were 5′-GCCTCTTTCCTTTACTTCTC-3′ and 5′-GGGTGTATTGAGCCTGATA-3′, respectively. An amplification reaction was carried out in 25 μL volumes consisting of 12.5 μL of 2xTaq PCR mix buffer (Sangon Biotech Co., Ltd, Shanghai, China), 1 μL of 100 ng/μL DNA template, 1 μL of 10 mM of each primer, and 9.5 μL of sterile ultrapure water. The PCR amplification procedure was as follows: predenaturation at 94 °C for 3 min, 36 cycles of amplification (94 °C 40 s, 58 °C 55 s, 72 °C 1 min), extension at 72 °C for 10 min, and finally preservation at 4 °C. The PCR products were bidirectionally sequenced by Sangon Biotech Co., Ltd.

### Data analysis

The D-loop sequences were separately aligned and trimmed to equal lengths using the MEGA 5.2 (Tamura et al. 2011) and ClustalX 2.1 (Thompson et al. 1997) software. Genetic diversity parameters were estimated using DnaSP 5.0 (Rozas et al. 2003) software. The Unweighted Pair Group Method with Arithmetic Mean (UPGMA) tree and the genetic distance among all the populations were constructed and computed using MEGA 5.2 software. Network 4.6 (Polzin and Daneshmand 2003) was used to construct Median Joining (MJ) network.

## Results

### Genetic diversity

A total of 350 homologous sequences of 907 bp were used for the genetic diversity analysis. A total of 28 nucleotide variation sites and 30 haplotypes (Hap) were detected in all individuals (Table 1). The haplotype diversity (*H_d_*) of ‘bicolor’ type *C. argus* was 0.187–0.911, and the *H_d_* of ‘white’ type *C. argus* was in the middle (0.301–0.505). The nucleotide diversity (*P_i_*) and average nucleotide differential number (*K*) of ‘bicolor’ type *C. argus* (*P_i_*: 0.00066–0.00326, *K*:0.598–2.955) were higher than ‘white’ type *C. argus* (*P_i_*: 0.00033–0.00057, *K*:0.321–0.521). The Tajima’s D values showed that all the populations did not significantly deviate from neutral (*p* > 0.05).

**Table 1.** Genetic diversity parameters of two color morphs *C. argus*.

Population	‘White’ type *C. argus*			‘Bicolor’ type *C. argus*			
	RC	NJ	LS	DH	WH	JR	HZ
Sample size	50	50	50	50	50	50	50
*S*	1	2	1	5	18	5	14
*Hp*	2	3	2	3	17	3	12
*H_d_*(SD)	0.429 (0.053)	0.505 (0.038)	0.301 (0.070)	0.187 (0.071)	0.911 (0.021)	0.471 (0.051)	0.851 (0.033)
*P*_i_(SD)	0.00047 (0.00006)	0.00057 (0.00005)	0.00033 (0.00008)	0.00066 (0.00025)	0.00300 (0.00022)	0.00157 (0.00018)	0.00326 (0.00023)
*K*	0.429	0.521	0.301	0.598	2.722	1.426	2.955
Tajima’s D	1.236	0.297	0.4692	−1.126	−1.015	0.672	−0.165

*Note*: S: number of polymorphic (segregating) site; Hp: number of haplotype; Hd: haplotype (gene) diversity; Pi: nucleotide diversity; K: average number of nucleotide differences; SD: standard deviation.

### Genetic differentiation

The fixation indexes (*F_ST_*) of ‘white’ type *C. argus* ranged from −0.00571 to 0.07261 (Table 2, above diagonal), and that in ‘bicolor’ type *C. argus* was higher (0.21902–0.85314). The *F_ST_* among the three ‘white’ type *C. argus* populations was not significant (*p* > 0.05); but was significant among ‘bicolor’ type *C. argus* populations and between the two color morphs of *C. argus* (*p* < 0.001). The gene flow (*Nm*) values among ‘bicolor’ type *C. argus* were less than 1, and the *Nm* absolute values among ‘white’ type *C. argus* were more than 1 (Table 2, below diagonal).

**Table 2.** *Nm* (below diagonal) and *F*_ST_ (above diagonal) of two color morphs *C. argus*.

Population	‘White’ type *C. argus*			‘Bicolor’ type *C. argus*			
	RC	NJ	LS	DH	WH	JR	HZ
RC	–	−0.00571 (*p* = 0.459946)	0.01909 (*p* = 0.16216)	0.83866 (*p* = 0.00000)	0.33241 (*p* = 0.00000)	0.64333 (*p* = 0.00000)	0.60286 (*p* = 0.00000)
NJ	−44.03283	–	0.07261 (*p* = 0.05405)	0.82952 (*p* = 0.00000)	0.34080 (*p* = 0.00000)	0.63946 (*p* = 0.00000)	0.60139 (*p* = 0.00000)
LS	12.84586	3.19305	–	0.85314 (*p* = 0.00000)	0.32507 (*p* = 0.00000)	0.65174 (*p* = 0.00000)	0.60672 (*p* = 0.00000)
DH	0.04809	0.05137	0.04303	–	0.31518 (*p* = 0.00000)	0.49428 (*p* = 0.00000)	0.34809 (*p* = 0.00000)
WH	0.50208	0.48356	0.51906	0.54319	–	0.21902 (*p* = 0.00000)	0.25562 (*p* = 0.00000)
JR	0.13860	0.14095	0.13358	0.25578	0.89144	–	0.38864 (*p* = 0.00000)
HZ	0.16468	0.16570	0.16205	0.46820	0.72801	0.39326	–

### Phylogenetic relationship analysis

**Figure 2.**
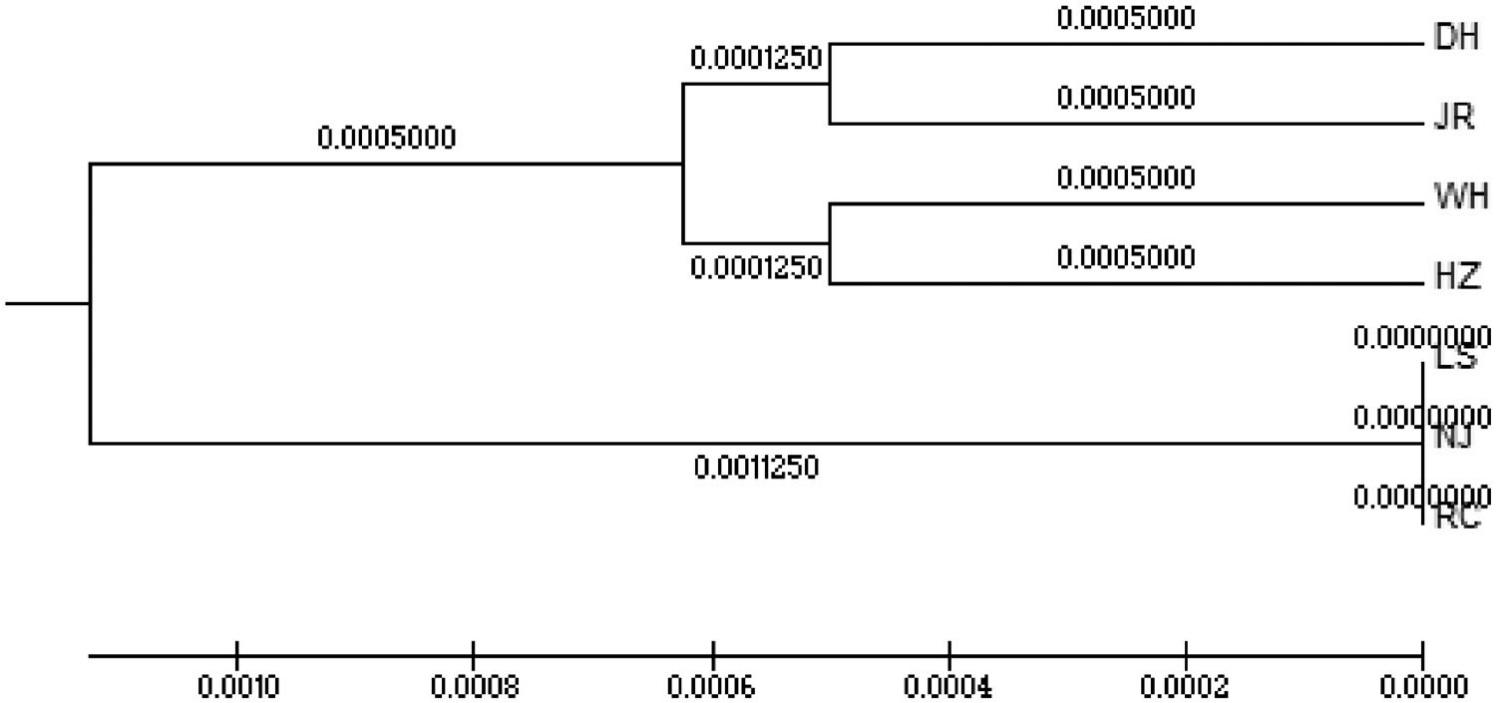
UPGMA tree of two color morphs *C. argus*.

The phylogenetic tree was inferred from these sequences using the Bootstrap method of UPGMA and MEGA 5.2 software. Two independent branches are shown in Figure 2; All the three ‘white’ type *C. argus* populations were clustered in one independent branch, and the genetic distance was very close.

### Haplotype network analysis

**Figure 3.**
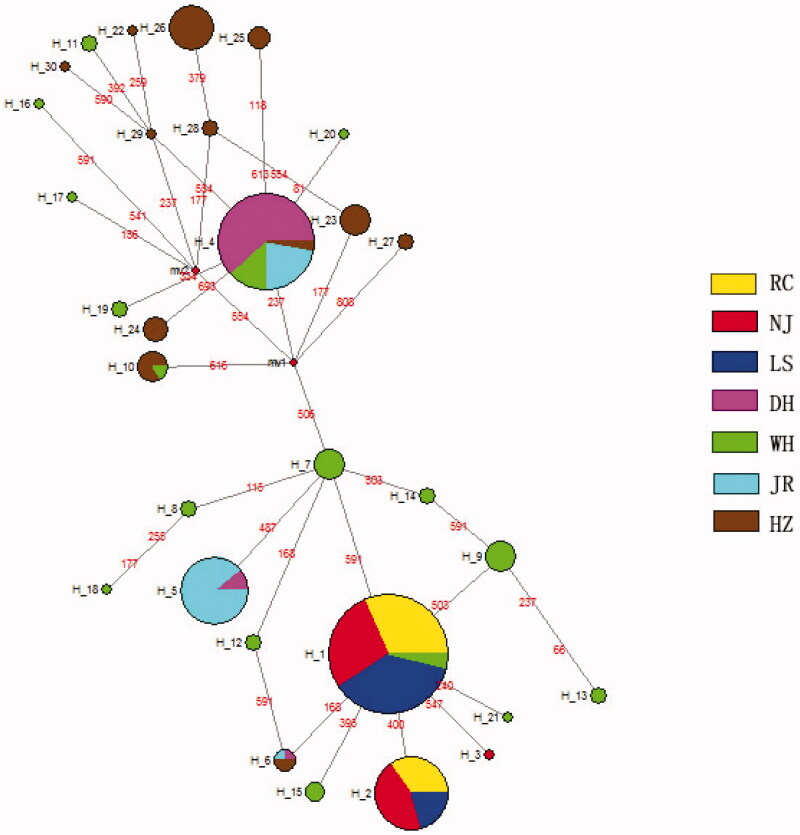
Haplotypes network of two color morphs *C. argus*.

The median joining (MJ) network was constructed for the identified 30 haplotypes (Figure 3). Only three haplotypes (Hap1, Hap2, and Hap3) existed in the three ‘white’ type *C. argus* populations; Hap1 and Hap2 were the dominant haplotypes in all the ‘white’ type *C. argus* populations, and Hap2 was not present in ‘bicolor’ type *C. argus*.

## Discussion

Haplotype diversity and nucleotide diversity are important indicators of population genetic variation (Wang et al. 2021). According to the range of *H_d_* and *P_i_* proposed by Grant and Bowen (1998), *H_d_* < 0.5 and *P_i_* < 0.005 showed slight genetic divergence across. In this study, the NJ ‘white’ type *C. argus* population (*H_d_* = 0.505), the WH and HZ ‘bicolor’ type *C. argus* populations (*H_d_* = 0.911, *H_d_* = 0.851, respectively) showed higher haplotype diversity, and the other populations showed lower haplotype diversity (*H_d_* < 0.5). The nucleotide diversity in all the two color morphs of *C. argus* was low (*P_i_* < 0.005), indicating slight genetic divergence across. However, the *P*_i_ and *K* of ‘bicolor’ type *C. argus* (*P_i_*: 0.00066–0.00326, *K*: 0.598–2.955) were higher than that in ‘white’ type *C. argus* (*P_i_*: 0.00033–0.00057, *K*: 0.321–0.521); the genetic diversity level of ‘white’ type *C. argus* populations was lower than ‘bicolor’ type *C. argus* populations. Few new parents introduced, thus decreasing the population genetic diversity. The lower genetic diversity is less tolerant to environmental stress (Zhou et al. 2017); the adaptive ability of ‘white’ type *C. argus* populations to the environment is weaker than that of ‘bicolor’ type *C. argus* populations.

*F_st_* is an important indicator of genetic differentiation among populations, 0 < *F_st_* < 0.05 indicates no differentiation; 0.05 < *F_st_* < 0.15 indicates moderate differentiation; 0.15 < *F_st_* < 0.25 indicates high differentiation (Wang et al. 2021). In this study, the *F_st_* of four ‘bicolor’ type *C. argus* populations was 0.21902–0.85314 (*p* < 0.01); moderate differentiation was observed between the WH and JR populations (*F_st_* = 0. 21902, 0.15 < *F_st_* < 0.25); high differentiation was observed among the other ‘bicolor’ type *C. argus* populations (*F_st_* was 0.25562 to 0.85314, *F_st_* > 0.25), indicating that the alleles on mitochondrial DNA D-loop region were differentiated and fixed in four ‘bicolor’ type *C. argus* populations. The *F_st_* of three ‘white’ type *C. argus* populations was −0.00571, 0.01909, and 0.07261 (*p* > 0.05); no significant differentiation was observed among the ‘white’ type *C. argus* populations. *Nm* < 1 between populations indicates that the population might be differentiated due to genetic drift; *Nm* > 1 indicates that the level of gene flow between populations was higher, and the genetic differentiation between populations was smaller (Wang et al. 2021). In this study, the values of *Nm* among the four ‘bicolor’ type *C. argus* populations were less than 1, indicating that geographic isolation had completely hindered the gene exchange of the four populations, but the absolute values of *Nm* among the three ‘white’ type *C. argus* populations were more than 1, indicating that the level of gene flow among those populations was high.

Genetic distance can be used to analyze the degree of genetic differentiation among different populations. The UPGMA phylogenetic tree indicated that a closer relationship was maintained in the ‘white’ type *C. argus* populations. The MJ network showed that the haplotype number in ‘white’ type *C. argus* was 10% of all the haplotypes; the genetic diversity of ‘white’ type *C. argus* was less than ‘bicolor’ type *C. argus*. The results of this study showed that the genetic diversity of ‘white’ type *C. argus* populations was relatively low. Therefore, attention should be paid to protecting the genetic diversity of ‘white’ type *C. argus* and to avoid inbreeding.

In this study, the genetic diversity and population structure were evaluated based on the complete sequences of mitochondrial DNA D-loop region for three cultured ‘white’ type *C. argus* populations and four ‘bicolor’ type *C. argus* populations, providing a theoretical basis for breeding, fishery resource protection, and sustainable development of *C. argus*. However, the genetic diversity and population structure of wild ‘white’ type *C. argus* populations were not analyzed in this study, and the genetic background could not be completely revealed. The studies should continue to use the mitochondrial DNA D-loop region of wild ‘white’ type *C. argus* populations to completely reveal the genetic background of ‘white’ type *C. argus* and to provide a theoretical basis for fishery resource protection in the future.

## Author contributions statement

Wei Fan and Lu Zhang, Conceptualization, Data analysis and interpretation, Writing original draft, Writing review and revising; Su Jian, Yu Luo, and Xiao-Lei Jiao, Conceptualization, Data analysis, Validation, Writing review and editing; Zhi-Peng Huang, Han Zhao, and Zhong-Meng Zhao, Data curation, Data analysis, Writing review and editing; Yuan-Liang Duan, Qiang Li, and Jun Du, Formal analysis, Writing review and editing; Ting Zhuo, Quan-Sen Su, and Jun Wu, Conceptualization, Writing review and revising; Jian Zhou, Conceptualization, Data curation, Supervision, Funding acquisition, Writing original draft, Revising it critically for intellectual content; and the final approval of the version to be published; and that all authors agree to be accountable for all aspects of the work.

## Ethical statement

This study was approved by the Institutional Animal Care and Use committee of the Neijiang Academy of Agricultural Sciences, Neijiang, Sichuan, China, under permit no. NAS-S20210501. All experiments were carried out in accordance with the Guide for the Care and Use of Experimental Animals of China.

## Data Availability

The data that support the findings of this study were available in figshare at https://doi.org/10.6084/m9.figshare.14872083. The sequence data that support the findings of this study are openly available in GenBank of NCBI at (https://www.ncbi.nlm.nih.gov/) under the accession no. MZ450074-MZ450103.
